# Effect of training in hypoxia on repeated sprint performance in female athletes

**DOI:** 10.1186/s40064-015-1041-4

**Published:** 2015-07-02

**Authors:** Nobukazu Kasai, Sahiro Mizuno, Sayuri Ishimoto, Etsuko Sakamoto, Misato Maruta, Kazushige Goto

**Affiliations:** Graduate School of Sport and Health Science, Ritsumeikan University, Kusatsu, Shiga Japan; Faculty of Sport and Health Science, Ritsumeikan University, 1-1-1 Nojihigashi, Kusatsu, Shiga 525-8577 Japan

**Keywords:** Normobaric hypoxia, Repeated sprint ability, Anaerobic power output, Team sport athletes

## Abstract

**Background:**

This study determined the effect of repeated sprint training in hypoxia (RSH) in female athletes.

**Methods:**

Thirty-two college female athletes performed repeated cycling sprints of two sets of 10 × 7-s sprints with a 30-s rest between sprints twice per week for 4 weeks under either normoxic conditions (RSN group; F_i_O_2_, 20.9%; n = 16) or hypoxic conditions (RSH group; F_i_O_2_, 14.5%; n = 16). The repeated sprint ability (10 × 7-s sprints) and maximal oxygen uptake ($\dot{\text{V}}{\text{O}}_{2\hbox{max} }$) were determined before and after the training period.

**Results:**

After training, when compared to pre-values, the mean power output was higher in all sprints during the repeated sprint test in the RSH group but only for the second half of the sprints in the RSN group (*P* ≤ 0.05). The percentage increases in peak and mean power output between before and after the training period were significantly greater in the RSH group than in the RSN group (peak power output, 5.0 ± 0.7% vs. 1.5 ± 0.9%, respectively; mean power output, 9.7 ± 0.9% vs. 6.0 ± 0.8%, respectively; *P* < 0.05). $\dot{\text{V}}{\text{O}}_{2\hbox{max} }$ did not change significantly after the training period in either group.

**Conclusion:**

Four weeks of RSH further enhanced the peak and mean power output during repeated sprint test compared with RSN.

## Background

The application of training in hypoxia for exercise capacity improvement has been accepted widely among endurance athletes (e.g., long-distance runners and swimmers), and a large amount of experimental evidence supports the efficacy of this training method (Dufour et al. 2006; Millet et al. 2010; Strzala et al. 2011). In contrast, growing evidence suggests that training in hypoxia also improves anaerobic or repeated sprints performance (Mizuno et al. 1990; Hamlin et al. 2010; Faiss et al. 2013a, 2015; Gatterer et al. 2014). Mizuno et al. (1990) revealed that 2 weeks of training at altitude significantly increased the time to exhaustion (TTE) in highly trained cross-country skiers during incremental treadmill running, with no change in maximal oxygen uptake ($\dot{\text{V}}{\text{O}}_{2\hbox{max} }$). Furthermore, improvements in running duration following the training period were significantly correlated with an increase in the buffering capacity of the gastrocnemius muscle. Moreover, intensive training in hypoxia (F_i_O_2_, 17.0–14.0%) for 10 successive days caused significantly greater increases in the mean power output during 30 s of maximal pedaling than did the same training in normoxia (Hamlin et al. 2010). Therefore, sprint training in hypoxia may further improve the anaerobic power output and sprint capacity (Billaut et al. 2012).

Team sports such as football, hockey, and basketball require athletes to perform a number of short sprints separated by periods of rest or low-to-moderate–intensity exercise (Bishop and Edge 2006). Recent studies have focused on the influences of repeated sprint training in hypoxia (RSH) on repeated sprint ability (Faiss et al. 2013a; Galvin et al. 2013; Millet et al. 2013; Gatterer et al. 2014). Faiss et al. (2013a) showed that 4 weeks of RSH further increased the number of sets until fatigue during a repeated sprint test (repeated 10-s maximal sprints with 20-s active recovery until exhaustion) compared with repeated sprint training in normoxia (RSN) among male cyclists. Furthermore, Gatterer et al. (2014) suggested that 5 weeks of shuttle running under hypoxia attenuated the reduction in sprint time during a shuttle run test (6 × 40-m sprints with 20-s passive recovery) compared with the same training in normoxia among young male soccer players. Although these findings suggest that RSH enhances anaerobic performance, further research is required using team sport athletes (Girard et al. 2013). In addition, the efficacy of RSH has been shown in male but not female team sport players (Galvin et al. 2013; Brocherie et al. 2015a, b). Because arterial O_2_ desaturation has been shown to be less sensitive to hypoxic stimuli in females than in males (Billaut and Smith 2009), the effect of RSH might be small in female athletes. Therefore, this study determined the effect of 4 weeks of RSH on the repeated sprint ability of female team sport athletes.

## Methods

### Subjects

Thirty-two college female athletes participated in this study (Table 1). All athletes were born and living at sea level. They belonged to the lacrosse club at the same university and performed lacrosse-specific training 5 days per week (2 h/day). This study was conducted during the basic training phase in a periodized training program.

**Table 1:** Physical characteristics and baseline measures of performance of the subjects in two groups

	RSH group	RSN group
Age (years)	20 ± 0.2	20 ± 0.1
Height (cm)	158.9 ± 1.4	158.9 ± 1.3
Body weight (kg)	53.9 ± 1.2	54.6 ± 1.6
Repeated sprint test		
Peak power (W/kg)	6.8 ± 0.1	6.9 ± 0.1
Mean power (W/kg)	5.6 ± 0.1	5.6 ± 0.1
Fatigue index (%)	12.2 ± 1.6	13.9 ± 1.5

Values are mean ± SE.

## Experimental design

The experiment began with a familiarization session with sprint training (under normoxia) before the baseline measurement. The baseline measurement was then made at least 48 h after the familiarization session. Based on the results of repeated sprint test during the baseline measurements, all subjects were assigned randomly to either the training group in normoxia (RSN group, n = 16) or the training group in hypoxia (RSH group, n = 16), matching the exercise capacity and physical characteristics (Table 1). No significant difference between the two groups was observed at the start of the training. All training sessions were conducted in an environmentally controlled chamber using an electromagnetically braked cycle ergometer (Power Max VIII; Konami Corporation, Tokyo, Japan). The subjects performed repeated pedaling with maximal effort twice per week for 4 weeks (eight training sessions in total) under normoxic conditions (F_i_O_2_, 20.9%) or hypoxic conditions (F_i_O_2_, 14.5%, equivalent to a simulated altitude of 3,000 m). This study was conducted in a single-blind fashion, and no subject was given information about group classification. The temperature in the chamber was maintained at 20°C during all training sessions. The hypoxic chamber was the whole-room type, and the hypoxic condition was established by insufflation of nitrogen (Morishima et al. 2013, 2014a; Morishima and Goto 2014). The oxygen and carbon dioxide concentrations within the chamber were continuously monitored.

**Figure 1:**
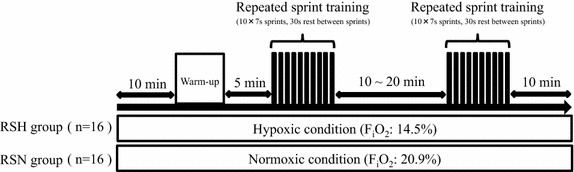
Protocol for each training session.

A standardized warm-up (30 s of submaximal sprinting and 2 × 3-s maximal sprinting) was performed on an ergometer. Each training session comprised two successive sets of 10 × 7-s sprints (maximal pedaling) with a 30-s rest period between sprints. The training protocol was designed in accordance with published protocols to enhance anaerobic performance and muscular adaptation (Edge et al. 2005; Faiss et al. 2013b). The physiological target for the training was improvements in the repeated sprint ability and buffer capacity (Edge et al. 2005; Buchheit and Laursen 2013). The load during pedaling corresponded to 4.0% of the subject’s body weight. The rest period between the two sets was set at 20 min during the first 2 weeks, and it was shortened to 10 min during the subsequent 2 weeks (Figure 1). Each training session was started after a 10-min rest following entrance into the chamber, and the subjects remained in the chamber for 10 min after completing the final set of training. Therefore, the total duration of exposure to hypoxia or normoxia was approximately 60 min in each training session. During all training sessions, the percutaneous oxygen saturation (SpO_2_) was monitored using a finger pulse oximeter (Smart Pulse; Fukuda Denshi, Tokyo, Japan) placed on the tip of the right forefinger. Before and after the training period, the repeated sprint ability (10 × 7-s sprints with a 30-s rest period between sprints) and maximal oxygen uptake ($\dot{\text{V}}{\text{O}}_{2\hbox{max} }$) were determined under normoxic conditions.

### Measuring maximal oxygen uptake and repeated sprint ability

The subjects visited the laboratory twice before the start of the 4-week training program. On the first visit, the subjects’ $\dot{\text{V}}{\text{O}}_{2\hbox{max} }$ was assessed using a graded power test on an ergometer (Aerobike 75XLIII; Konami Corporation, Tokyo, Japan). Before the graded power test, the subjects performed a standardized warm-up (pedaling at 50 W for 5 min). The test began at 70 W, and the load was increased progressively by 35-W increments every 2 min until exhaustion (70 rpm). The TTE was also evaluated during the test. The TTE was defined as the exercise duration until the subjects failed to maintain a pedaling frequency of 70 rpm for 5 s. For the graded power test, the first criterion of exhaustion was maintenance of pedaling frequency. In addition to the first criterion, when the two of four criteria (1.$\dot{\text{V}}{\text{O}}_{2\hbox{max} }$ plateau, 2. respiratory exchange ratio ≥1.10, 3. Heart rate (HR) reaching at least 90% of the theoretical maximal HR, 4. rating of perceived exertion ≥19) were fulfilled, the test was terminated (Howley et al. 1995). Respiratory gases were collected and analyzed using an automatic gas analyzer (AE300S; Minato Medical Science, Tokyo, Japan). The collected data were averaged every 30 s. Each subject’s HR during exercise was measured continuously using a wireless HR monitor (RS400; Polar Electro, Tokyo, Japan). The rating of perceived exertion was determined every 2 min using a Borg 15-point scale (Borg 1973).

On the second visit, the subjects conducted a repeated sprint test under normoxic conditions, comprising 10 × 7-s sprints (maximal pedaling) with a 30-s rest period between sprints. Before the tests, the subjects completed a standardized warm-up (30 s of submaximal pedaling and 2 × 3-s sprint) on an electromagnetically braked cycle ergometer (Power Max VIII; Konami Corporation, Tokyo, Japan). The applied load for the repeated sprint test was equivalent to 4.0% of the subject’s body weight. The peak and mean power outputs during each sprint were recorded. The fatigue index was calculated as the magnitude of the percentage reduction over 10 sprints [(sprint 1 − sprint 10)/sprint 1] × 100 (Glaister et al. 2008). After the training period, a graded power test and repeated sprint test were performed to assess $\dot{\text{V}}{\text{O}}_{2\hbox{max} }$.

### Statistical analysis

All data are expressed as mean ± standard error (SE). To compare power output during the repeated sprint test, two-way analysis of variance (ANOVA) with repeated measures was applied to confirm the interaction [training period (before, after training period) × number of sprints (sprints 1 − 10)] or main effect (training period, number of sprints). When ANOVA revealed a significant interaction or main effect, the Tukey–Kramer post hoc test was performed to identify differences. To compare $\dot{\text{V}}{\text{O}}_{2\hbox{max} }$ and TTE between before and after the training period, two-way ANOVA with repeated measures [group (RSH, RSN group), training period (before, after training period)] was performed. Percentage changes in the power output during the repeated sprint test (relative to pretraining values) were compared between the two groups using an unpaired *t* test. For all tests, *P* < 0.05 was considered to indicate statistical significance.

## Results

### Physiological and performance variables during the training period

The average values of SpO_2_ during all training sessions were significantly lower in the RSH group (92.5 ± 0.3%) than in the RSN group (97.7 ± 0.4%, *P* < 0.05). Body weight did not change significantly after the training period in the RSH group (before, 53.9 ± 1.2 kg; after, 54.3 ± 1.2 kg; n.s.) or the RSN group (before, 54.6 ± 1.6 kg; after, 54.6 ± 1.6 kg; n.s.).

During the 4-week training period, both groups showed increases in power output during the training sessions, but the temporal changes differed between the groups. In the RSH group, significant increases in the peak power output (relative to the value for the first training session) were observed during training sessions 4 and 6–8 (*P* < 0.05), whereas the RSN group did not show a significant increase relative to the first training session over all training sessions. During sessions 3, 4, and 6–8, the relative values of the peak power output were significantly higher in the RSH group than in the RSN group (*P* < 0.05). Both groups showed significant increases in the mean power output during the eight training sessions. However, the RSH group showed a significant increase in the relative value of the mean power output in session 4, whereas the RSN group showed a significant increase in session 8.

### Performance during the repeated sprint test

**Figure 2:**
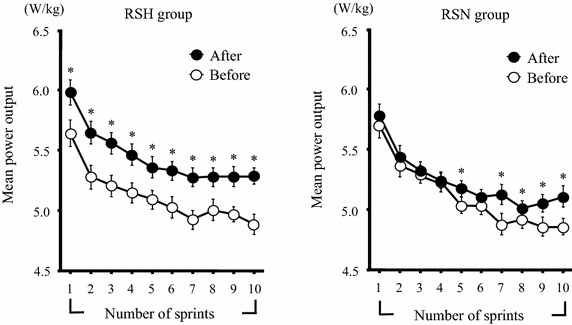
Time-course changes in the mean power output during the repeated sprint test in the RSH and RSN groups before and after the training period. Values are mean ± SE. *P < 0.05 vs. corresponding value before the training period.

Figure 2 presents the mean power output during the repeated sprint test before and after the training period. The mean power output during each sprint decreased significantly with increased numbers of sprints in both groups (*P* < 0.05). In the RSH group, significant increases (relative to the pretraining values) in the mean power output were observed in all sprints (*P* < 0.05), whereas the RSN group showed significant increases in sprints 5 and 7–10 (*P* < 0.05).

**Figure 3:**
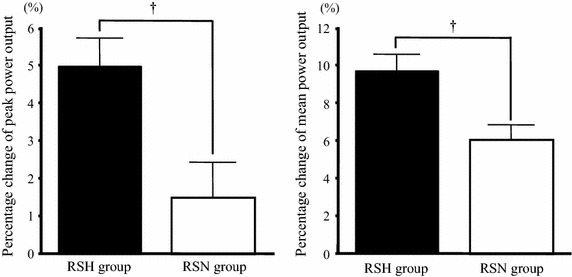
Percentage changes in the peak and mean power outputs during the repeated sprint test before and after the training period. Values are mean ± SE. ^†^ *P* < 0.05 between the RSH and RSN groups.

When the magnitude of the percentage changes (relative to pretraining values) in the power output during the repeated sprint test was compared between the two groups (Figure 3), the RSH group showed a significantly greater percentage increase in peak power output than did the RSN group (5.0 ± 0.7% vs. 1.5 ± 0.9%, respectively; *P* < 0.05). In addition, the RSH group showed a significantly greater percentage increase in the average values of mean power output over 10 sprints than did the RSN group (9.7 ± 0.9% vs. 6.0 ± 0.8%, respectively; *P* < 0.05). When the fatigue index during the repeated sprint test was compared between before and after the training period, the RSN group showed a significant reduction in the fatigue index after the training period (before, 13.9 ± 1.5%; after, 9.6 ± 1.1%; *P* < 0.05). The RSH group also showed a tendency toward a reduction after the training period (before, 12.2 ± 1.6%; after, 9.2 ± 0.9%; *P* = 0.05).

### $\dot{\text{V}}{\text{O}}_{2\hbox{max} }$ and time to exhaustion

There were no significant differences in $\dot{\text{V}}{\text{O}}_{2\hbox{max} }$ or maximal HR between the two groups before the training period (Table 2). These values were not significantly different in either group between before and after the training period. However, the RSH group showed a significant increase in TTE after the training period (*P* < 0.05), whereas no significant change was observed in the RSN group. The maximal HR did not change significantly after the training period in either group.

**Table 2:** Respiratory variables and performance during the graded power test

	Before	After
$$ \dot{\text{V}}{\text{O}}_{2\hbox{max} } $$ (ml/min)		
RSH group	2,398 ± 52	2,470 ± 59
RSN group	2,461 ± 79	2,473 ± 67
$\dot{\text{V}}{\text{O}}_{2\hbox{max} }$/BW (ml/min/kg)		
RSH group	44.6 ± 0.8	45.9 ± 0.7
RSN group	45.1 ± 1.0	45.4 ± 0.8
Time to exhaustion (s)		
RSH group	659 ± 18	688 ± 21*
RSN group	656 ± 23	678 ± 22
Maximal HR (bpm)		
RSH group	194 ± 2	193 ± 1
RSN group	190 ± 2	189 ± 2

Values are mean ± SE.

* P < 0.05 between before and after the training period.

## Discussion

This study compared the effects of 4 weeks of repeated sprint training between a training group in hypoxia (RSH group) and a training group in normoxia (RSN group). The results indicated that the RSH group showed significantly greater improvement in repeated sprint ability than did the RSN group. A novel finding of this study was that an approximately three-fold greater increase in peak power output during repeated sprint test was observed in the RSH group than in the RSN group. To our knowledge, this is the first report to show that high-intensity training under hypoxic conditions is beneficial for improving maximal power output.

Both groups showed significant increases in the mean power output during the repeated sprint test after the 4-week training period. However, when we compared the relative increase from the pretraining values between the two groups, the RSH group showed a significantly greater percentage change than did the RSN group. This result suggests that the RSH group had further improvement in the repeated sprint ability compared with the RSN group. These findings are supported by recent publications. Hamlin et al. (2010) reported that sprint training in moderate hypoxia (F_i_O_2_, 17.0–14.0%) for 10 successive days caused significantly greater increases in the mean power output during 30 s of maximal pedaling than the same training in normoxia. Faiss et al. (2013a) also showed that 4 weeks of RSH (3 sets of 5 × 10-s sprint; F_i_O_2_, 14.6%) resulted in a significant increase in the number of sets until exhaustion during the repeated sprint test, whereas no change was found after the same training in normoxia. Galvin et al. (2013) recently reported that 4 weeks of RSH (F_i_O_2_, 13.0%) resulted in two-fold greater improvements in distance during the intermittent running test than did equivalent RSN. The repeated sprint ability is related to the capacity for phosphocreatine (PCr) resynthesis (Mendez-Villanueva et al. 2012) and aerobic capacity (Edge et al. 2005; Bishop et al. 2011). Although we were unable to evaluate the capacity for PCr resynthesis, $\dot{\text{V}}{\text{O}}_{2\hbox{max} }$ (an indicator of aerobic capacity) did not change significantly in either group after the training period. During short-duration, high-intensity exercise of <60 s, the metabolic response under hypoxia has been suggested to differ from that under normoxia, independent of similar exercise capacities (Weyand et al. 1999). Two studies (Ogura et al. 2006; Ogawa et al. 2007) indicated that the power output during 40 s of maximal pedaling or a maximal anaerobic running test did not differ between hypoxic and normoxic conditions. However, the contribution of the anaerobic energy supply was augmented by about 9.3% in hypoxia compared with normoxia (Ogura et al. 2006). The augmented energy supply from the anaerobic system might cause greater stimulus for adaptation and further improvement in the repeated sprint ability.

The novel finding of this study was that the power output during the first sprint in the repeated sprint test increased significantly only in the RSH group. In the repeated sprint test, a significant increase in power output during all sprints was observed in the RSH group, whereas the RSN group showed significant increases during the second half of the repeated sprint exercise. However, growing evidence suggests that RSH and high-intensity interval training under hypoxic conditions produce greater improvements in the mean power output (anaerobic endurance capacity) (Hamlin et al. 2010; Faiss et al. 2013a, b; Galvin et al. 2013). Our finding of further improvement in the power output of the first sprint has not been reported. During 7-s maximal pedaling, the ATP-PCr system rather than the glycolytic system is thought to play a main role in ATP production. Therefore, the improved power output during the first sprint in the RSH group might be related to an augmented PCr content in muscle. In support of this hypothesis, we recently observed that six consecutive days of RSH significantly increased the PCr content in muscle among sprinters (unpublished observation). Further research involving determination of the intramuscular PCr content is necessary.

In this study, we monitored the time course of the changes in power output during all training sessions in each group. Consequently, the RSH group showed a significantly greater increase in power output during the second week (versus the value in first training session), whereas a similar increase was observed during fourth week in the RSN group. Therefore, the training stimulus might be higher in the RSH group during the training period, leading to greater adaptation of sprint performance. Furthermore, the fatigue index was reduced significantly after the training period in the RSN group alone. However, caution is necessary when interpreting this later result because the absence of significant improvement in the RSH group is thought to be due to marked enhancement of the power output in the first sprint during repeated sprint test.

A unique point of this study is that we recruited female team sport athletes. Although we found marked improvements in repeated sprint ability, we cannot conclude whether the training effects in this study are specific to female athletes because of a lack of comparative data from males. Because female athletes tend to have lower anaerobic power outputs than do male athletes (Brooks et al. 1990; Billaut and Smith 2009), there might be greater trainability in response to sprint training. In contrast, females have been shown to be less sensitive to hypoxic stimuli for arterial O_2_ desaturation (Billaut and Smith 2009). Therefore, it is unlikely that our results are specific to female athletes. We believe that RSH is suitable for both male and female athletes.

In this study, twice-weekly repeated sprint training sessions (approximately 60 min per training session) were incorporated into the regular training schedule. Therefore, the training protocol is practical and would be suitable for most athletes. Our finding suggests that RSH is a new strategy for augmenting the maximal anaerobic power output and repeated sprint ability in female athletes. This study focused on the adaptation of sprint performance, and future studies are necessary to reveal the detailed mechanism behind the adaptation.

## Conclusion

Four weeks of RSH caused further increases in the peak and mean power outputs during the repeated sprint test compared with RSN in female team sport athletes.
